# Simple method for detecting idiopathic interstitial pneumonias by measuring vertical lung length on chest X-ray

**DOI:** 10.1038/s41598-021-87452-z

**Published:** 2021-04-07

**Authors:** Masato Karayama, Yoichiro Aoshima, Hideki Yasui, Hironao Hozumi, Yuzo Suzuki, Kazuki Furuhashi, Tomoyuki Fujisawa, Noriyuki Enomoto, Yutaro Nakamura, Naoki Inui, Takafumi Suda

**Affiliations:** 1 Second Division, Department of Internal Medicine, Hamamatsu University School of Medicine, 1-20-1 Handayama, Hamamatsu, 431-3192 Japan; 2 Department of Clinical Pharmacology and Therapeutics, Hamamatsu University School of Medicine, 1-20-1 Handayama, Hamamatsu, 431-3192 Japan

**Keywords:** Respiratory tract diseases, Diagnosis, Radiography

## Abstract

Detection of idiopathic interstitial pneumonias (IIPs) on chest X-ray is difficult for non-specialist physicians, especially in patients with mild IIPs. The current study aimed to evaluate the usefulness of a simple method for detecting IIPs by measuring vertical lung length (VLL) in chest X-rays to quantify decreased lung volume. A total of 280 consecutive patients with IIPs were randomly allocated to exploratory and validation cohorts, and 140 controls were selected for each cohort by propensity score-matching. Upper (*u*VLL; from apex to tracheal carina), lower (*l*VLL; from carina to costophrenic angle), and total VLL (*t*VLL; from apex to costophrenic angle), and the *l*/*u*VLL ratio were measured on chest X-rays. Patients in the exploratory cohort had significantly decreased *u*VLL, *l*VLL, *t*VLL, and *l*/*u*VLL ratio compared with controls (all *p* < 0.001). Receiver operating characteristic curve analyses demonstrated that *l*VLL (area under the curve [AUC] 0.86, sensitivity 0.65, specificity 0.92), *t*VLL (AUC 0.83, sensitivity 0.75, specificity 0.80), and *l*/*u*VLL ratio (AUC 0.80, sensitivity 0.72, specificity 0.79) had high diagnostic accuracies for IIPs. These results were reproduced in the validation cohort. IIP patients thus have decreased VLLs, and measurements of VLL may thus aid the accurate detection of IIPs.

## Introduction

Idiopathic interstitial pneumonias (IIPs) are progressive fibrotic diseases of the lungs with unknown etiology^1^. Although their precise prevalence is unclear, IIPs are not uncommon and idiopathic pulmonary fibrosis (IPF), as the main subtype of IIPs, was estimated to account for up to 65,000 s death in Europe and up to 17,000 in the United States in 2014^2^, while an epidemiologic survey in Japan reported a prevalence of IPF of 10.0 per 100,000 population and a median survival time of 35 months^3^. Although IIPs have been poorly treated in the past, early intervention has become increasingly important over the last decade in line with the emergence of anti-fibrotic agents^4–7^, and routine screening in primary care practice is expected to improve the early detection of IIPs.

IIPs have also become clinically important for non-pulmonologists in relation to the increasing use of biological, molecular targeted, and immune checkpoint-inhibitor agents as standard therapies in various fields of clinical medicine. Despite their efficacy, these novel treatments have raised concern about the occurrence of drug-induced interstitial lung diseases as a life-threatening toxicity^8–11^. Pre-existing interstitial pneumonias, such as IIPs, are recognized as a major risk factor for drug-induced interstitial lung disease, and patients should thus be screened for interstitial pneumonia before administering these therapies^8,9,12,13^.

Radiologic imaging plays an important role in the screening and diagnosis of IIPs. Honeycombing, reticular shadows, and ground-grass opacity are typical radiologic features of IIPs^1,14–17^, and chest computed tomography (CT), especially high-resolution CT, is the gold standard for evaluating these features. However, the risk of radiation exposure and/or overuse of medical resources mean that routine chest CT screening of IIPs is not always practicable, especially in developing countries, where access to CT examinations is sometimes poor because of problems with medical resources and insurance systems.

Decreased lung volume, resulting from pulmonary fibrosis, is another feature of IIPs^16,18^ and can be confirmed by reduced lung fields and an elevated diaphragm on chest X-ray. However, precise evaluation of IIP-related pulmonary abnormalities on chest X-ray is difficult for non-pulmonologists, such as primary physicians or oncologists, and even well-trained pulmonologists and radiologists may not find it easy to detect early-stage IIPs on chest X-ray^18,19^. In contrast, reduced lung fields and an elevated diaphragm are easily evaluable and quantifiable on chest X-rays, even by doctors without expertise in diagnostic radiology, by measuring the vertical length of the lung. However, whether or how decreased lung volume is quantified on chest X-rays in patients with IIPs remain unclear. In the present study, we aimed to develop a method to quantify decreased lung volume by measuring the vertical lung length (VLL) on chest X-ray, and to evaluate its usefulness for the detection of IIPs.

## Results

### Patient characteristics

**Figure 1:**
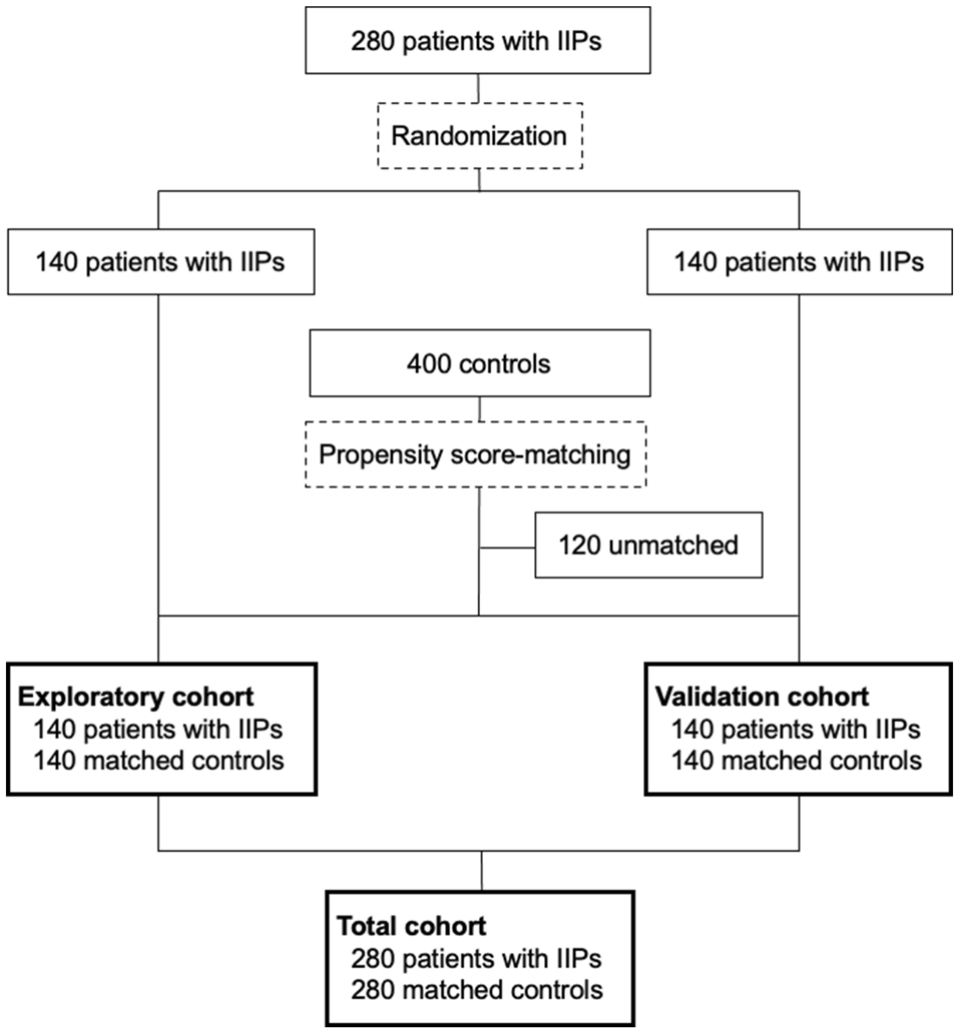
Diagram of the study. Patients with idiopathic interstitial pneumonias (IIPs) were randomized, stratified according to sex and %predicted forced vital capacity. Controls were selected by one-to-one propensity score-matching using age, sex, and body mass index.

The current study included an exploratory and validation cohort, each comprising 140 patients with IIPs and 140 propensity-score matched control subjects (Fig. 1). The patient characteristics are presented in Table 1. A total of 137 patients (48.9%) had > 80% of %predicted forced vital capacity (FVC), indicating mild IIP. The patients with IIPs and matched control subjects in each cohort had comparable characteristics, except in terms of spirometry results. The exploratory and validation cohorts had comparable characteristics. The most dominant IIP subtype was IPF (74.2%). All of the patients with IIPs had a reticular pattern on chest CT images, and 70.4% and 94.3% had honeycombing and ground-glass opacity (GGO), respectively (Table 2).

**Table 1:** Patient characteristics.

	Exploratory cohort		Validation cohort		Total cohort	
	Controls, n = 140	IIPs, n = 140	Control, n = 140	IIPs, n = 140	Controls, n = 280	IIPs, n = 280
**Age**	70.5 (31–93)	70 (41–92)	71.0 (24–88)	69 (36–85)	71.0 (24–93)	70 (36–92)
**Sex, male**	107 (76.4)	107 (76.4)	107 (76.4)	107 (76.4)	214 (76.4)	214 (76.4)
**Body-mass index**	22.4 (16.5–48.0)	23.1 (16.2–29.0)	23.4 (14.7–33.1)	22.7 (15.6–35.6)	22.9 (14.7–48.1)	23.0 (15.6–35.6)
**Spirometry**						
%predicted FVC	99.6 (80.9–132.2)	78.8 (28.0–122.0)*	100.2 (79.9–137.1)	79.9 (30.0–132.8)*	100.0 (79.9–137.1)	79.3 (28.0–132.8)*
%predicted FEV_1_	95.8 (77.9–141.4)	81.0 (34.8–130.6)*	98.1 (78.7–135.6)	84.5 (36.0–141.1)*	97.1 (77.9–141.4)	81.4 (34.8–141.1)*
FEV_1_/FVC ratio	78.0 (70.1–105.1)	81.5 (47.9–100.0)*	79.4 (70.1–116.3)	84.4 (57.0–99.0)*	79.0 (70.1–116.3)	83.2 (47.9–100.0)*
**Subtypes of IIPs**						
IPF		104 (74.3)		104 (74.3)		208 (74.2)
Fibrotic NSIP		6 (4.3)		8 (5.7)		14 (5.0)
Unclassifiable		19 (13.6)		16 (11.4)		35 (12.5)

Data presented as median (range) or number (%).

*FEV*_*1*_ forced expiratory volume in 1s, *FVC* forced vital capacity, *IIPs* idiopathic interstitial pneumonias, *IPAF* interstitial pneumonia with autoimmune features, *IPF* idiopathic pulmonary fibrosis, *NSIP* non-specific interstitial pneumonia.

*p < 0.001 compared with control subjects in each cohort.

**Table 2:** Findings of chest computed tomography in patients with idiopathic interstitial pneumonias.

	Exploratory cohort, n = 140	Validation cohort, n = 140	Total cohort, n = 280
Reticular pattern, grade 0/1/2/ ≥ 3	0 (0)/94 (67.1)/32 (22.9)/14 (10.0)	0 (0)/92 (65.7)/42 (30.0)/6 (4.3)	0 (0)/186 (66.4)/74 (26.4)/20 (7.2)
Honeycombing, grade 0/1/2/ ≥ 3	34 (24.3)/84 (60.0)/18 (12.9)/4 (2.8)	49 (35.0)/71 (50.7)/15 (10.7)/5 (3.6)	83 (29.6)/155 (55.4)/33 (11.8)/9 (3.3)
GGO, grade 0/1/2/ ≥ 3	10 (7.1)/122 (87.1)/8 (5.7)/0 (0)	6 (4.3)/112 (80.0)/ 20 (14.3)/2 (1.4)	16 (5.7)/234 (83.6)/28 (10.0)/2 (0.7)
%LAA	10.7 (1.0–48.5)	15.1 (1.0–52.0)	12.8 (1.0–52.0)

Data presented as median (range) or number (%). The extent of chest CT findings were semi-quantitatively evaluated as follows: grade 0 (0%), grade 1 (< 25%), grade 2 (25–50%), grade 3 (50–75%), and grade 4 (> 75%).

*GGO* ground-glass opacity, *%LAA* percentage low-attenuation area.

### VLLs in patients with IIPs and controls in the exploratory cohort

**Figure 2:**
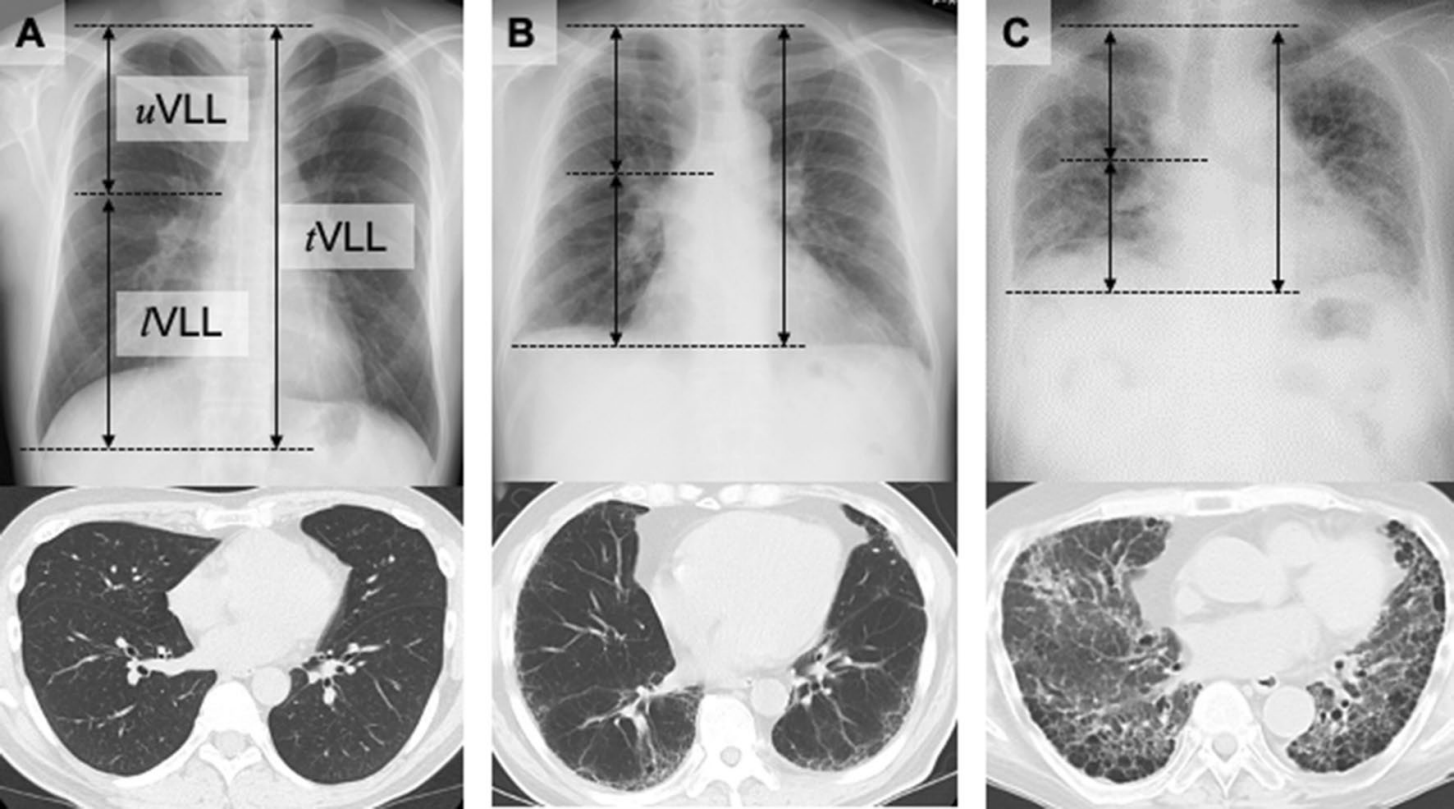
Measurements of vertical lung length (VLL) on chest X-rays. Three VLL measurements were taken: upper VLL (*u*VLL), from the apex to the costophrenic angle; lower VLL (*l*VLL), from the tracheal carina to the costophrenic angle; and total VLL (*t*VLL), from the apex to the costophrenic angle. Measurements were performed in the right lung. Compared with control subjects (**A**), patients with mild interstitial pneumonia (IIP) (**B**) and severe IIP (**C**) demonstrated decreased *u*VLL, *l*VLL, and *t*VLL. The decrease in *l*VLL was especially notable.

VLL was measured on chest X-rays as follows: (1) from the apex to the costophrenic angle (total VLL, *t*VLL); (2) from the apex to the carina of the trachea (upper VLL, *u*VLL); and (3) from the carina to the costophrenic angle (lower VLL, *l*VLL) (Fig. 2A–C). The VLLs were adjusted by body height [VLLs (mm/m) = unadjusted VLLs (mm)/body height (m)]. The intraclass correlation coefficients for tVLL, uVLL, lVLL, and the l/uVLL ratio in the total cohort were 0.924, 0.890, 0.893, and 0.827, respectively.

**Figure 3:**
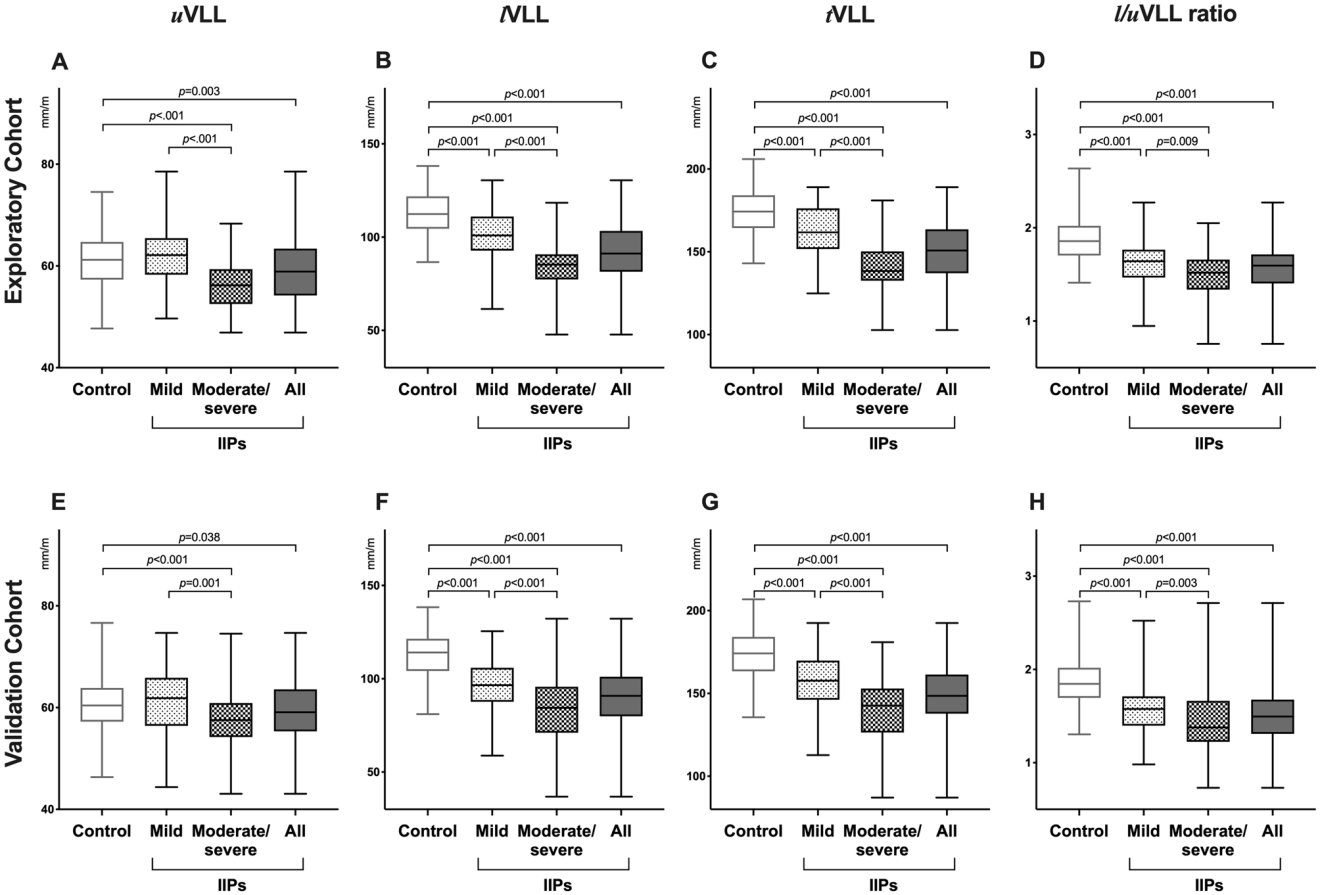
Comparison of vertical lung lengths (VLLs) between patients with IIPs and controls. VLLs in the exploratory (**A**–**D**) and validation cohorts (**E**–**H**). Mild and moderate/severe idiopathic interstitial pneumonias (IIPs) were defined as patients with %predicted forced vital capacity ≥ 80% and < 80%, respectively. White, black, light grey, and grey boxes represent controls, all patients with IIPs, mild IIPs, and moderate/severe IIPs, respectively.

In the exploratory cohort, patients with IIPs had significantly lower *u*VLL, *l*VLL, *t*VLL, and *l*/*u*VLL ratio compared with the control subjects (*p* = 0.003, < 0.001, < 0.001, and < 0.001, respectively) (Fig. 3A–D, Supplementary Table 1). Patients with IIPs were grouped into mild IIPs (%predicted FVC ≥ 80%) and moderate/severe IIPs (< 80%). Patients with moderate/severe IIPs had significantly lower *u*VLL, *l*VLL, *t*VLL, and *l*/*u*VLL ratio compared with those with mild IIPs and control subjects (all *p* < 0.001, except *p* = 0.009 for *l*/*u*VLL ratio for mild IIPs). In addition, patients with mild IIPs also had significantly lower *l*VLL, *t*VLL, and *l*/*u*VLL ratio, but not *u*VLL, compared with the control subjects (all *p* < 0.001, except *u*VLL).

### Receiver operating characteristic (ROC) analyses of VLL for detection of IIPs in the exploratory cohort

**Figure 4:**
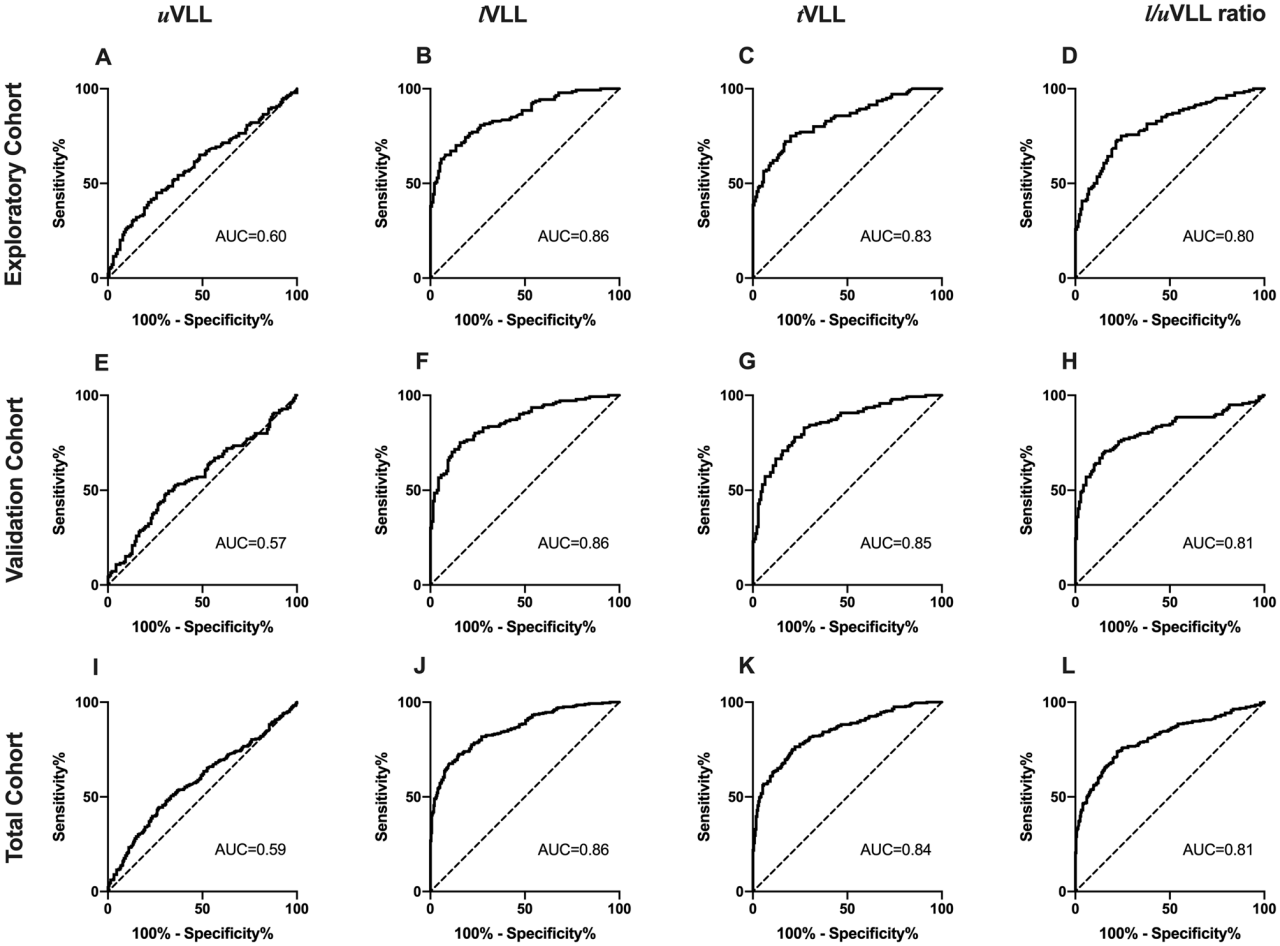
Receiver operating characteristic curves of vertical lung lengths (VLLs) for the detection of IIPs. Receiver operating characteristic curve analyses of VLLs in the exploratory (**A**–**D**), validation (**E**–**H**), and total cohorts (**I**–**L**). *AUC* area under the curve.

ROC analyses demonstrated high diagnostic accuracies of *l*VLL (area under the curve [AUC] 0.86), *t*VLL (AUC 0.83), and *l*/*u*VLL ratio (AUC 0.80), but not *u*VLL (AUC 0.60) for the detection of IIPs (Fig. 4A–D).

**Figure 5:**
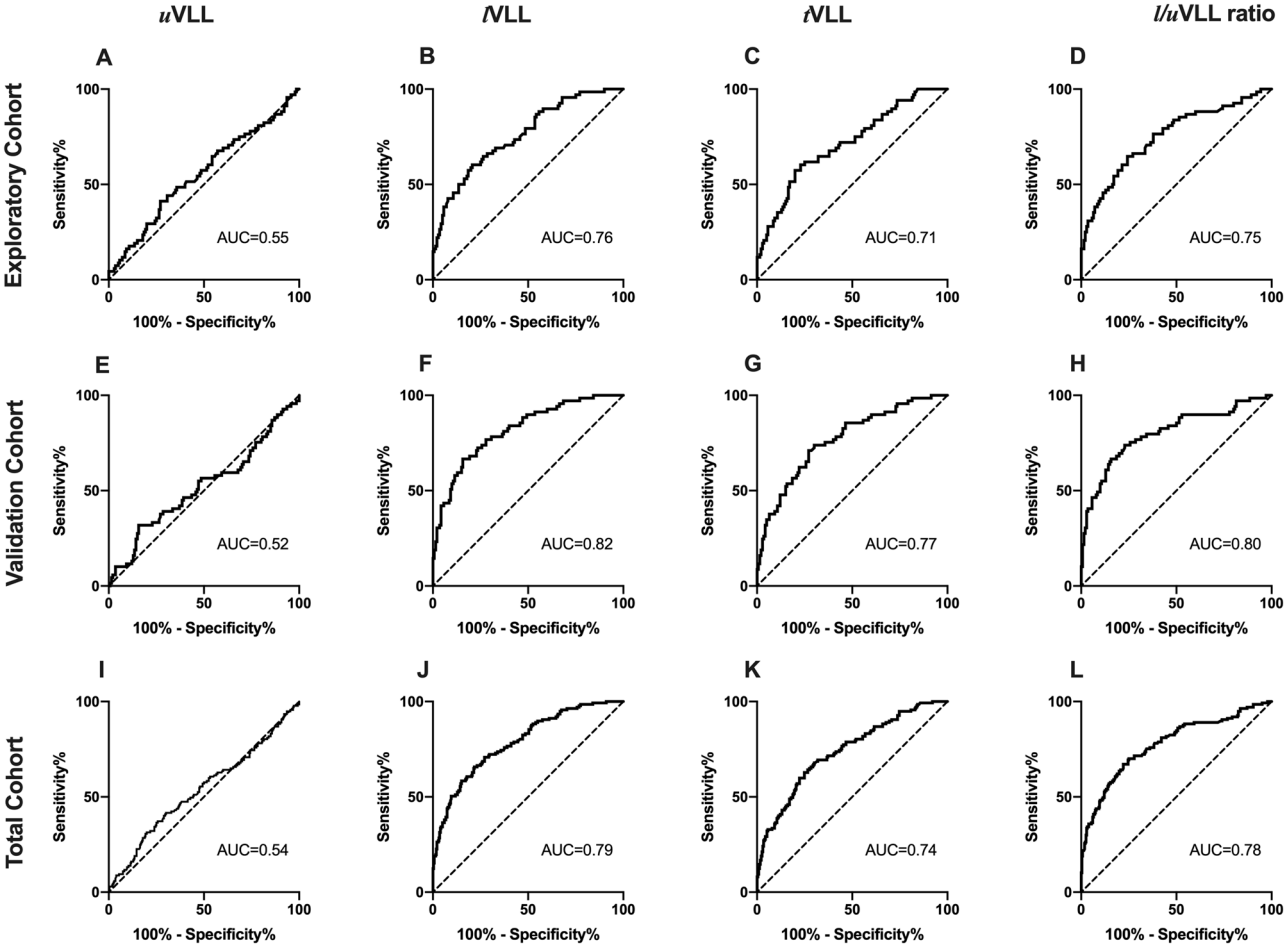
Receiver operating characteristic curves of vertical lung lengths (VLLs) for the detection of mild IIPs. Receiver operating characteristic curve analyses of VLLs for the detection mild IIPs (%predicted forced vital capacity ≥ 80%) in the exploratory (**A**–**D**), validation (**E**–**H**), and total cohorts (**I**–**L**). *AUC* area under the curve.

Cut-off values were 98 mm/m for *l*VLL (sensitivity 0.65, specificity 0.92), 163 mm/m for *t*VLL (sensitivity 0.75, specificity 0.80), and 1.68 for *l*/*u*VLL ratio (sensitivity 0.72, specificity 0.79). When limited to patients with mild IIPs, *l*VLL, *t*VLL, and *l*/*u*VLL ratio, but not *u*VLL, also demonstrated high diagnostic accuracies (AUCs 0.76, 0.71, 0.75, and 0.55, respectively) (Fig. 5A–D). Cut-off values were 103 mm/m for *l*VLL (sensitivity 0.60, specificity 0.79), 164 mm/m for *t*VLL (sensitivity 0.60, specificity 0.77), and 1.70 for *l*/*u*VLL ratio (sensitivity 0.65, specificity 0.76).

### Validation of differences in VLLs and cut-off values in validation cohort

The differences in VLLs between patients with IIPs and the control subjects were reproduced in the validation cohort. Patients with IIPs had significantly lower *u*VLL, *l*VLL, *t*VLL, and *l*/*u*VLL ratio compared with the control subjects (*p* = 0.038, < 0.001, < 0.001, and < 0.001, respectively) (Fig. 3E–H, Supplementary Table 1). Patients with moderate/severe IIPs also had significantly lower *u*VLL, *l*VLL, *t*VLL, and *l*/*u*VLL ratio compared with patients with mild IIPs and control subjects (all *p* < 0.001, except *p* = 0.001 and 0.003 for *u*VLL and *l*/*u*VLL ratio for mild IIP, respectively). Patients with mild IIPs had significantly lower *l*VLL, *t*VLL, and *l*/*u*VLL ratio, but not *u*VLL, compared with the control subjects (all *p* < 0.001, except *u*VLL).

The AUCs of *u*VLL, *l*VLL, *t*VLL, and *l*/*u*VLL ratio for the detection of IIPs were comparable to those in the exploratory cohort (0.57, 0.86, 0.85, and 0.81, respectively) (Fig. 4E–H). The cut-off values for the detection of IIPs determined in the exploratory cohort demonstrated reproducible diagnostic accuracies in the validation cohort (*l*VLL: sensitivity 0.71, specificity 0.87; *t*VLL: sensitivity 0.78, specificity 0.78; *l*/*u*VLL ratio: sensitivity 0.76, specificity 0.77).

Regarding patients with mild IIPs, the AUCs of *u*VLL, *l*VLL, *t*VLL, and *l*/*u*VLL ratio were also comparable to those in the exploratory cohort (0.52, 0.82, 0.77, and 0.80, respectively) (Fig. 5E–H), and the cut-off values determined in the exploratory cohort demonstrated reproducible diagnostic accuracies in the validation cohort (*l*VLL: sensitivity 0.79, specificity 0.76; *t*VLL: sensitivity 0.79, specificity 0.74; and *l*/*u*VLL ratio: sensitivity 0.75, specificity 0.74).

### Final cut-off values in total cohort

The final cut-off values for the detection of IIPs in the total cohort were 98 mm/m for *l*VLL (sensitivity 0.68, specificity 0.90, AUC 0.86), 163 mm/m for *t*VLL (sensitivity 0.76, specificity 0.79, AUC 0.84), and 1.68 for *l*/*u*VLL ratio (sensitivity 0.74, specificity 0.78, AUC 0.81) (Fig. 4I–L). The AUC for *l*VLL was significantly higher than those for uVLL (*p* < 0.001), *t*VLL (*p* = 0.030), and *l*/*u*VLL ratio (*p* < 0.001). There was no significant difference in AUCs between *t*VLL and *l*/*u*VLL ratio.

When limited to patients with mild IIPs, the final cut-off values in the total cohort were 103 mm/m for *l*VLL (sensitivity 0.66, specificity 0.78, AUC 0.79), 164 mm/m for *t*VLL (sensitivity 0.63, specificity 0.76, AUC 0.74), and 1.70 for *l*/*u*VLL ratio (sensitivity 0.70, specificity 0.75, AUC 0.78) (Fig. 5I–L). The AUC for *l*VLL was significantly higher than those for *u*VLL (*p* < 0.001) and *t*VLL (*p* < 0.001). There was no significant difference in AUCs between *l*VLL and *l*/*u*VLL ratio, or between *t*VLL and *l*/*u*VLL ratio.

When using the VLL cutoff values in round numbers in clinical practice, an lVLL of 100 mm/m had a sensitivity of 0.71 and specificity of 0.86 for all IIPs, and a sensitivity of 0.54 and specificity of 0.86 for mild IIPs. A tVLL of 160 mm/m had a sensitivity of 0.70 and specificity of 0.83 for all IIPs, and a sensitivity of 0.49 and specificity of 0.83 for mild IIPs. An l/uVLL ratio of 1.70 had a sensitivity of 0.76 and a specificity of 0.75 for all IIPs, and a sensitivity of 0.70 and specificity of 0.75 for mild IIPs.

### Correlations of VLLs with clinical factors and findings of chest computed tomography

**Figure 6:**
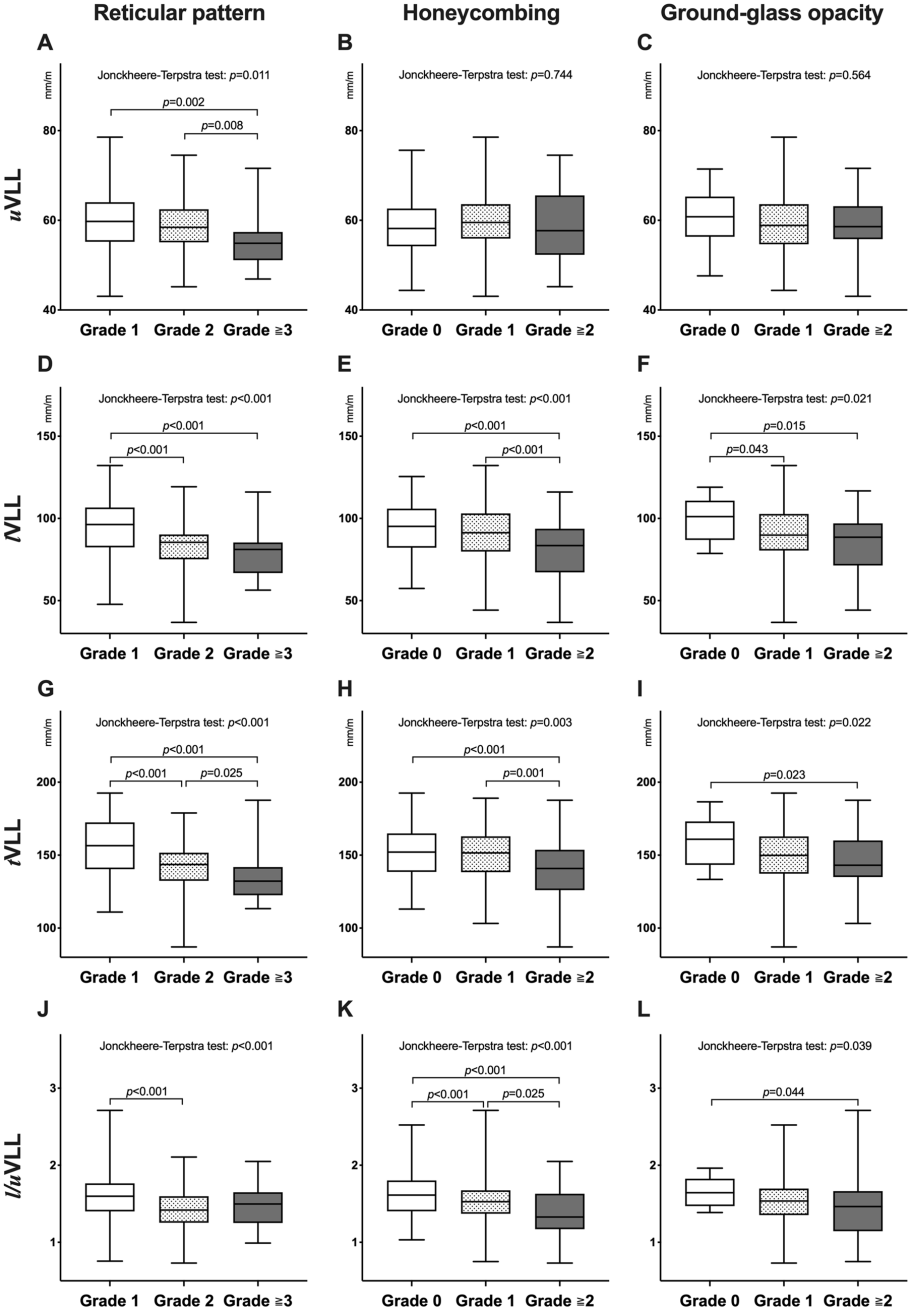
Associations of vertical lung lengths (VLLs) with the extent of lung abnormalities on chest CT images. The association of reticular pattern, honeycombing, and ground-glass opacity with upper VLL (uVLL) (**A**–**C**), lower VLL (lVLL) (**D**–**F**), total VLL (tVLL) (**G**–**I**), and l/uVLL ratio (**J**–**L**) are shown. The extents of the lung abnormalities were semi-quantitatively evaluated as follows: grade 0 (0%), grade 1 (< 25%), grade 2 (25–50%), grade 3 (50–75%), and grade 4 (> 75%). The white, grey, and black boxes in the images represent grade 1, 2, and ≥ 3 for reticular pattern, respectively, or grade 0, 1, and ≥ 2 for honeycombing and ground-glass opacity, respectively.

The *t*VLL and *l*VLL showed moderate positive correlations with %predicted FVC (*r* = 0.63 and 0.60, respectively) and %predicted forced expiratory volume in 1 s (FEV_1_) (*r* = 0.54 and 0.53, respectively), and weak inverse correlations with body mass index (BMI) (*r* =  − 0.31 and − 0.29, respectively) and FEV_1_/FVC ratio (*r* =  − 0.27 and − 0.23, respectively) (Table 3). The *u*VLL and *l*/*u*VLL had weak positive correlations with %predicted FVC (*r* = 0.30 and 0.41, respectively) and %predicted FEV_1_ (*r* = 0.22 and 0.38, respectively), and weak inverse correlations with FEV_1_/FVC ratio (*r* =  − 0.21 and − 0.10, respectively). The uVLL was negatively associated with the extent of the reticular pattern, but not with honeycombing or GGO (*p* = 0.011, 0.744, and 0.564, respectively) (Fig. 6A–C). lVLL, tVLL, and l/uVLL were negatively associated with the extent of the reticular pattern (all *p* < 0.001), honeycombing (*p* < 0.001, 0.003, and < 0.001, respectively), and ground-glass opacity (GGO) on chest computed CT images (*p* = 0.021, 0.022, and 0.039, respectively) (Fig. 6D–L). The patients with honeycombing (n = 190) had significantly lower lVLL, tVLL, and l/uVLL values than those without (n = 90) (*p* = 0.005, 0.020, and 0.001, respectively). When compared with the controls, both patients with and without honeycombing had significantly lower uVLL (*p* = 0.003 and 0.006, respectively), lVLL (both *p* < 0.001), tVLL (both *p* < 0.001), and l/uVLL (both *p* < 0.001) values. Percentage low-attenuation area (%LAA), defined by the percentage area below − 950 HU in the total lung area on chest CT images had weak positive correlations with uVLL, lVLL, and tVLL (r = 0.24, 0.38, and 0.34, respectively) and a very weak correlation with the l/uVLL (r = 0.19) (Table 3). After adjusting for age, sex, BMI, %predicted FVC, %predicted FEV_1_, and %LAA in multivariate analyses, *l*VLL, *t*VLL, and *l*/*u*VLL ratio were identified as independent predictive factors for the detection of IIPs and mild IIPs (Tables 4 and 5).

**Table 3:** Correlations between vertical lung length and clinical factors.

	Age	Body mass index	%Predicted FVC	%Predicted FEV_1_	FEV_1_/FVC	%LAA
*u*VLL	0.06 (0.13)	− 0.17 (< 0.01)	0.30 (< 0.01)	0.22 (< 0.01)	− 0.21 (< 0.01)	0.24 (< 0.01)
*l*VLL	0.01 (0.86)	− 0.29 (< 0.01)	0.60 (< 0.01)	0.53 (< 0.01)	− 0.23 (< 0.01)	0.34 (< 0.01)
*t*VLL	0.03 (0.52)	− 0.31 (< 0.01)	0.63 (< 0.01)	0.54 (< 0.01)	− 0.27 (< 0.01)	0.38 (< 0.01)
*l*/*u*VLL ratio	− 0.06 (0.20)	− 0.17 (< 0.01)	0.41 (< 0.01)	0.38 (< 0.01)	− 0.10 (0.02)	0.19 (< 0.01)

Data expressed as Pearson’s correlation coefficient (*p* value).

*FEV*_*1*_ forced expiratory volume in 1s, *FVC* forced vital capacity, *%LAA* percentage low-attenuation area.

**Table 4:** Logistic regression analyses for idiopathic interstitial pneumonias.

Variables	Odds ratio	*p* value	Odds ratio	*p* value	Odds ratio	*p* value	Odds ratio	*p* value
Age	0.99 (0.96–1.01)	0.361	0.99 (0.97–1.01)	0.391	0.99 (0.96–1.01)	0.337	0.98 (0.96–1.01)	0.264
Sex, male	1.31 (0.69–2.51)	0.408	0.78 (0.42–1.43)	0.417	1.09 (0.56–2.10)	0.807	0.70 (0.36–1.33)	0.274
BMI	0.83 (0.76–0.91)	< 0.001	0.98 (0.92–1.05)	0.569	0.82 (0.75–0.89)	< 0.001	0.91 (0.85–0.98)	0.013
FVC_%pred_	0.95 (0.91–0.98)	< 0.001	0.91 (0.88–0.93)	< 0.001	0.94 (0.91–0.97)	< 0.001	0.91 (0.88–0.94)	< 0.001
FEV_1%pred_	0.99 (0.96–1.02)	0.536	1.00 (0.97–1.03)	0.778	1.00 (0.97–1.03)	0.910	1.00 (0.97–1.04)	0.819
%LAA	0.98 (0.96–1.00)	0.109	0.97 (0.96–0.99)	0.011	0.98 (0.96–1.00)	0.078	0.98 (0.96–1.00)	0.024
*t*VLL	0.93 (0.91–0.95)	< 0.001						
*u*VLL			1.03 (0.99–1.06)	0.144				
*l*VLL					0.90 (0.88–0.93)	< 0.001		
*l*/*u*VLL ratio							2.12 × 10^–2^ (0.76 × 10^–2^–0.60 × 10^–1^)	< 0.001

Data expressed as odds ratio (95% confidence interval). Odds ratios for continuous variables expressed as per 1 value increase.

*BMI* body mass index, *FEV*_*1%pred*_ percentage predicted forced expiratory volume in 1s, *FVC*_*%pred*_ percentage predicted forced vital capacity, *tVLL* total vertical lung length, *uVLL* upper vertical lung length, *lVLL* lower vertical lung length, *%LAA* percentage low-attenuation area.

**Table 5:** Logistic regression analyses for mild idiopathic interstitial pneumonias.

Variables	Odds ratio	*p* value	Odds ratio	*p* value	Odds ratio	*p* value	Odds ratio	*p* value
Age	0.99 (0.96–1.02)	0.397	0.99 (0.97–1.02)	0.507	0.98 (0.96–1.01)	0.280	0.99 (0.96–1.01)	0.304
Sex, male	1.40 (0.72–2.71)	0.324	0.70 (0.38–1.30)	0.266	1.21 (0.61–2.40)	0.589	0.69 (0.35–1.35)	0.277
BMI	0.84 (0.77–0.92)	< 0.001	1.01 (0.94–1.08)	0.815	0.82 (0.74–0.90)	< 0.001	0.93 (0.86–1.00)	0.038
FVC_%pred_	0.97 (0.94–1.01)	0.132	0.94 (0.91–0.97)	< 0.001	0.97 (0.94–1.01)	0.123	0.95 (0.92–0.99)	0.007
FEV_1%pred_	0.99 (0.96–1.02)	0.523	0.99 (0.96–1.03)	0.725	1.00 (0.96–1.03)	0.849	1.00 (0.97–1.04)	0.986
%LAA	0.98 (0.96–1.01)	0.155	0.98 (0.96–1.00)	0.039	0.98 (0.96–1.00)	0.120	0.98 (0.96–1.00)	0.061
*t*VLL	0.93 (0.91–0.95)	< 0.001						
*u*VLL			1.05 (1.01–1.10)	0.016				
*l*VLL					0.90 (0.88–0.92)	< 0.001		
*l*/*u*VLL ratio							1.14 × 10^–2^ (0.34 × 10^–2^–0.38 × 10^–1^)	< 0.001

Data expressed as odds ratio (95% confidence interval). Odds ratios for continuous variables expressed as per 1 value increase.

*BMI* body mass index, *FEV*_*1%pred*_ percentage predicted forced expiratory volume in 1s, *FVC*_*%pred*_ percentage predicted forced vital capacity, *tVLL* total vertical lung length, *uVLL* upper vertical lung length, *lVLL* lower vertical lung length, *%LAA* percentage low-attenuation area.

### Subgroup analysis of patients with IPF and non-IPF

When patients with IIPs were divided into IPF and non-IPF groups, both groups demonstrated significantly lower *u*VLL (*p* = 0.003 and 0.005, respectively), *t*VLL (both *p* < 0.001), *l*VLL (both *p* < 0.001), and *l*/*u*VLL ratio (both *p* < 0.001) compared with the control subjects. Compared with the IPF group, non-IPF patients had significantly higher *l*VLL and *l*/*u*VLL ratio (*p* = 0.043 and 0.007, respectively), but similar *u*VLL and *t*VLL (*p* = 0.490 and 0.132, respectively). After adjusting for age, sex, BMI, %predicted FVC, %predicted FEV_1_, and %LAA in multivariate logistic regression analysis, there was no significant difference in *u*VLL, *l*VLL, *t*VLL, or *l*/*u*VLL ratio between the IPF and non-IPF groups (Supplementary Table 2).

### Secondary interstitial pneumonia cohort

Next, we evaluated 240 patients with secondary interstitial pneumonias (IPs). The secondary IPs comprised collagen vascular disease (CVD)-associated IPs (n = 116), sarcoidosis (n = 76), chronic hypersensitivity pneumonia (CHP) (n = 40), and pneumoconiosis (n = 8). The types of CVD were rheumatoid arthritis (n = 49), dermatomyositis (n = 26), Sjögren syndrome (n = 17), scleroderma (n = 8), systemic lupus erythematosus (n = 2), a combination of two or more CVDs (n = 5), and others (n = 14). Compared with the patients with IIPs, those with secondary IPs were significantly younger (*p* < 0.001), female-dominant (*p* < 0.001), and had lower BMI (*p* = 0.006), higher %predicted FVC, higher %predicted FEV_1_ (*p* < 0.001), and lower FEV_1_/FVC (*p* = 0.009) (Supplementary Table 3). The patients with secondary IPs demonstrated significantly lower *u*VLL (*p* = 0.014) and higher *l*VLL, *t*VLL, and *l*/*u*VLL ratio (all *p* < 0.001), compared with those with IIPs (Supplementary Table 4).

When limited to the patients with %predicted FVC ≥ 80%, patients with secondary IPs demonstrated significantly lower *u*VLL (*p* = 0.002) and higher *l*VLL, *t*VLL, and *l*/*u*VLL ratio (*p* < 0.001, 0.002, and < 0.001, respectively), compared with those with IIPs (Supplementary Table 4).

Compared with patients with IPF**,** those with CVD-IP had significantly higher *l*VLL (*p* < 0.001), *t*VLL (*p* = 0.001), and *l*/*u*VLL ratio (*p* < 0.001); those with sarcoidosis had significantly lower *u*VLL (*p* = 0.009) and higher *l*VLL (*p* < 0.001), *t*VLL (*p* < 0.001), and *l*/*u*VLL ratio (*p* < 0.001); those with CHP had significantly lower *u*VLL (*p* < 0.001) and higher *l*VLL (*p* < 0.001) and *l*/*u*VLL ratio (*p* < 0.001); and those with pneumoconiosis had significantly higher *l*VLL (*p* < 0.001), *t*VLL (*p* = 0.003), and *l*/*u*VLL ratio (*p* = 0.002).

## Discussion

In the present study, we evaluated reduced lung volume by measuring VLLs on chest X-rays, and found that VLLs were significantly decreased in patients with IIPs. Among the measured VLLs, *l*VLL, *t*VLL, and *l*/*u*VLL ratio demonstrated high diagnostic accuracies for the detection of IIPs, with *l*VLL having the highest accuracy. Importantly, decreases in *l*VLL, *t*VLL, and *l*/*u*VLL ratio were also observed in patients with mild IIPs, with a %predicted FVC > 80%. Chest X-rays are a non-invasive, low-radiation exposure, and low-cost diagnostic method, and the measurement of VLLs is simple and easy, even for doctors with no expertise in diagnostic radiology, thus aiding the early detection of IIPs in clinical practice.

Among the decreases in VLLs observed in patients with IIPs, the decrease in *l*VLL was more prominent compared with *u*VLL, possibly because pulmonary fibrosis occurs predominantly in the lower lobe of the lungs. Although the uneven distribution of pulmonary fibrosis in the lower lungs is well known in IPF^14–16,20^, the underlying mechanisms are unknown. One hypothesis suggests that mechanical stress caused by repeated ventilation is stronger in the lower lobe because of the large motion of the chest wall and diaphragm^20–22^. In addition to IPF, most IIPs demonstrate predominantly lower lobe lesions^16,19,20,23^. Patients in the current study with non-IPF IIPs showed decreased *l*VLL and *l*/*u*VLL ratio, similar to patients with IPF.

Interestingly, VLLs were decreased in patients with IIP but with no decline in %predicted FVC. In addition to lung elasticity, pulmonary function also depends on thoracic elasticity and the respiratory muscles, and patients with IPF were reported to show only a weak or moderate correlation between pulmonary function and radiologic severity at baseline and changes during follow-up^17,24^. In the present study, VLLs were only moderately correlated with %predicted FVC, and the decreases in VLLs were independent of clinical factors, including %predicted FVC. In addition, differences in the cut-off values between all IIPs and mild IIPs were small. These characteristics of VLLs thus enable the detection of early-stage IIPs, but conversely, VLLs may be of little clinical use for estimating the deterioration of pulmonary function.

Among the measured VLLs, although *l*VLL demonstrated the highest AUC for the detection of IIPs, the diagnostic accuracies of *l*VLL, *t*VLL, and *l*/*u*VLL ratio were similar from a clinical perspective. In contrast to *l*VLL and *t*VLL, which need to be adjusted for body height, *l*/*u*VLL ratio does not depend on height and can be calculated from chest X-ray images, and can thus be used as an alternative to *l*VLL.

The current study had three main limitations. First, VLLs are only evaluable on chest X-rays performed in an upright position, in patients with no abnormal changes other than interstitial pneumonias. Chest CT should be used to screen for IIPs in patients in the decubitus position, or those with a history of chest surgery or other obvious chest abnormalities. Similarly, severely obese patients may have an elevated diaphragm due to visceral fat and may therefore be unsuitable for the evaluation of VLLs. Second, the differential distribution of pulmonary fibrosis affects VLLs. Patients with secondary IPs demonstrated lower *u*VLL and higher *l*VLL than those with IIPs. This may be because secondary IPs, including sarcoidosis, CHP, and pneumoconiosis, demonstrate upper-lobe-predominant fibrosis, whereas IPF, the most dominant phenotype among IIPs, demonstrates lower-lobe-predominant fibrosis^25–27^. Furthermore, CVD-IPs are a heterogeneous group of different disease phenotypes, each with distinct lung abnormalities varying according to the phenotype. The utility of VLLs in secondary IPs needs to be investigated further. Third, the present study was retrospective in nature, with limited clinical information. The existence of respiratory symptoms, dyspnea, or abnormal respiratory sounds on auscultation may increase the diagnostic ability in combination with VLLs. The clinical utility of VLLs thus needs to be validated in a prospective, observational study in a real-world setting.

## Conclusions

Patients with IIPs, including those with mild IIPs, had decreased VLLs, especially *l*VLL, on chest X-ray. The measurements of *l*VLL, *t*VLL, and *l*/uVLL ratio demonstrated high diagnostic accuracies for the detection of IIPs. This simple method may aid the early detection of IIPs by non-respiratory practitioners and physicians in areas with poor access to CT examinations.

## Methods

### Study design

This retrospective observational study was conducted in accordance with the ethical standards described in the Declaration of Helsinki. The study protocol was approved by the Institutional Review Board of Hamamatsu University School of Medicine (No. 20-015). Each patient provided written informed consent to be included in the study. The study was registered with the University Hospital Medical Information Network Clinical Trial Registry (identification code: 000040724).

### Patient eligibility

We retrospectively evaluated the medical records of patients with IIPs in our institute from January 2000 to December 2019. The diagnosis of IIP was made according to American Thoracic Society/European Respiratory Society Clinical Practice Guidelines^1^. The inclusion criteria were as follows: chest X-ray and spirometry performed within 1 week of each other, and clinically stable disease with no worsening for at least 4 weeks before spirometry and chest X-ray. If multiple data were available for the same patient, the earliest data from the initial diagnosis of IIP were evaluated. The exclusion criteria were as follows: patients with pleuroparenchymal fibroelastosis, characterized by upper-lobe-dominant fibrosis^28^; patients with anatomical chest abnormalities, abnormal chest shadows without IIP (e.g. lung cancer, pleural effusion, or pneumothorax), or a history of chest surgery; patients with missing data for either chest X-ray or spirometry within 1 week; and patients with clinically unstable disease within 4 weeks (e.g. pneumonia or acute exacerbation). Clinically stable patients without IIPs were evaluated as control subjects. Control subjects were required to have normal spirometry, no pulmonary disease, no anatomical chest abnormality, and no history of chest surgery.

### Exploratory and validation cohorts

A total of 280 consecutive patients with IIPs and 400 control subjects were evaluated. The patients with IIPs were randomly allocated to the exploratory or validation cohort (Fig. 1). Control subjects were selected for each cohort by propensity score-matching using age, sex, and BMI. Each cohort finally included 140 patients with IIPs and 140 control subjects. The two cohorts were merged into a total cohort for final analysis.

### Secondary interstitial pneumonia cohorts

A total of 240 consecutive patients with secondary IPs, including connective tissue diseases, sarcoidosis, hypersensitivity pneumonia, and pneumoconiosis, were evaluated as the secondary IP cohort.

### Evaluation of chest X-rays and computed tomography

Lung volume loss was evaluated by measuring *u*VLL, *l*VLL, and *t*VLL in the right lung on chest X-rays taken in the upright position and at maximum inspiration (Fig. 2A–C). The data were adjusted for body height [VLLs (mm/m) = unadjusted VLLs (mm)/body height (m)]. The *l*/*u* VLL ratio was also calculated. The right (instead of the left) lung was evaluated to eliminate the possible effect of cardiac shadow. VLLs were evaluated by two independent investigators and the results are expressed as a mean of the two estimates. The extent of the reticular pattern, honeycombing, and GGO on chest CT images were semi-quantitatively evaluated as follows: grade 0 (0%), grade 1 (< 25%), grade 2 (25–50%), grade 3 (50–75%), and grade 4 (> 75%)^29^. The definitions of reticular pattern, honeycombing, and GGO were according to the Fleishner Society criteria^30^. The %LAA on chest CT images was calculated using image-analyzing software (SYNAPSE VINCENT; Fuji Film, Tokyo, Japan)*.*

### Statistical analyses

The randomization of patients with IIPs was stratified by sex and %predicted FVC. One-to-one propensity score-matching was performed using age, sex, and BMI as co-variables. Continuous variables were compared by Wilcoxon’s rank sum test and categorical variables by Fisher’s exact test. The predictive values of VLLs for the diagnosis of IIPs were evaluated by multivariate logistic regression analysis. The cut-off values of VLLs for the diagnosis of IIPs were estimated by ROC analysis and determined using Youden’s index (maximum value of [sensitivity + specificity − 1]). The correlations between VLLs and clinical factors were evaluated by Pearson’s correlation analysis. The correlations of VLLs with the increased extent of lung abnormalities in the patients with IIPs were evaluated by the Jonckheere–Terpstra test. The inter-observer reproducibility was evaluated by intraclass correlation analyses. A *p* value < 0.05 (two-sided) was considered significant. All statistical analyses were carried out using JMP v13.0.0 (SAS Institute Japan, Tokyo, Japan), except The Jonckheere–Terpstra test was performed using EZR (Saitama Medical Center, Jichi Medical University, Saitama, Japan), a graphical user interface for R (The R Foundation for Statistical Computing, Vienna, Austria).

## Supplementary Information

Supplementary Information.

## Data Availability

All data generated or analyzed during this study are included in this published article and Supplementary Information file.
