# Supplementary material for: Simple method for detecting idiopathic interstitial pneumonias by measuring vertical lung length on chest X-ray
Source: Sci Rep. 2021 Apr 7;11:7669. doi: 10.1038/s41598-021-87452-z (PMC8027863; doi:10.1038/s41598-021-87452-z)
Supplement: Supplementary file 1 — Supplementary Information. [file 41598_2021_87452_MOESM1_ESM.pdf]

Supplementary File

Simple method for detecting idiopathic interstitial pneumonias by measuring vertical lung length on chest X-ray

Authors: Masato Karayama[1*], Yoichiro Aoshima[1], Hideki Yasui[1], Hironao Hozumi[1], Yuzo Suzuki[1], Kazuki Furuhashi[1], Tomoyuki Fujisawa[1], Noriyuki Enomoto[1], Yutaro Nakamura[1], Naoki Inui[1,2], Takafumi Suda[1]

Affiliations:

[1]Second Division, Department of Internal Medicine, Hamamatsu University School of Medicine, 1-20-1 Handayama, Hamamatsu 431-3192, Japan

[2]Department of Clinical Pharmacology and Therapeutics, Hamamatsu University School of Medicine, 1-20-1 Handayama, Hamamatsu 431-3192, Japan

Supplementary File

Supplementary Table 1. Results of vertical lung length

	Exploratory cohort		Validation cohort		Total cohort	
	Controls, n=140	IIPs, n=140	Control, n=140	IIPs, n=140	Controls, n=280	IIPs, n=280
Unadjusted *u*VLL, mm	100 (69-131)	95 (68-130)#	98 (67-123)	94 (66-130)#	99 (67-131)	95 (66-130)*
*u*VLL, mm/m	61 (48-75)	59 (47-79)#	60 (46-77)	59 (43-75)#	61 (46-77)	59 (43-79)*
Unadjusted *l*VLL,mm	183 (134-233)	147 (74-227)*	184 (133-228)	143 (57-201)*	184 (133-233)	145 (57-227)*
*l*VLL, mm/m	112 (87-138)	91 (48-130)*	114 (81-138)	91 (37-125)*	113 (81-138)	91 (37-130)*
Unadjusted *t*VLL, mm	282 (215-358)	241 (151-327)*	282 (202-336)	238 (135-315)*	282 (202-358)	240 (135-327)*
*t*VLL, mm/m	164 (143-206)	137 (103-189)*	174 (136-207)	149 (87-193)*	174 (136-207)	150 (87-193)*
l/*u*VLL ratio	1.86 (1.41-2.64)	1.59 (0.76-2.27)*	1.85 (1.30-2.73)	1.49 (0.73-2.71)*	1.85 (1.30-2.73)	1.55 (0.73-2.71)*

Data presented as median (range).

IIPs, idiopathic interstitial pneumonias; *t*VLL, total vertical length of the lung; *u*VLL, upper vertical length of the lungs; *l*VLL, lower vertical length of the lung.

*$p<0.001$ compared with control subjects in each cohort.

#$p<0.005$ compared with control subjects in each cohort.

Supplementary Table 2. Logistic regression analyses for idiopathic pulmonary fibrosis and non-idiopathic pulmonary fibrosis

Variables	Odds ratio	*p*-value	Odds ratio	*p*-value	Odds ratio	*p*-value	Odds ratio	*p*-value
Age	1.03 (0.99-1.08)	0.088	1.04 (1.00-1.08)	0.074	1.03 (1.00-1.08)	0.085	1.03 (0.99-1.07)	0.089
Sex, male	6.62 (3.36-13.06)	<.001	6.35 (3.20-12.60)	<.001	6.36 (3.23-12.50)	<.001	6.06 (3.08-11.93)	<.001
BMI	0.96 (0.85-1.09)	0.566	1.01 (0.91-1.13)	0.807	0.97 (0.86-1.09)	0.598	0.97 (0.94-1.01)	0.886
$FVC_{\%pred}$	0.98 (0.95-1.02)	0.371	0.97 (0.94-1.01)	0.094	0.98 (0.95-1.02)	0.264	1.02 (0.98-1.05)	0.110
$FEV_{1\%pred}$	1.01 (0.98-1.05)	0.497	1.01 (0.98-1.05)	0.410	1.01 (0.98-1.05)	0.438	1.01 (0.98-1.04)	0.381
%LAA	1.01 (0.98-1.04)	0.402	1.01 (0.98-1.04)	0.539	1.01 (0.98-1.04)	0.441	0.50 (0.18-1.41)	0.528
*t*VLL	0.98 (0.96-1.01)	0.161						
*u*VLL			1.00 (0.95-1.05)	0.899				
*l*VLL					0.98 (0.96-1.01)	0.137		
*l*VLL/*u*VLL ratio							0.97 (0.94-1.01)	0.193

Data are expressed as odds ratio (95% confident interval) for idiopathic pulmonary fibrosis. In continuous variables, odds ratio is expressed as per 1 value increase. BMI, body mass index; $FEV_{1\%pred}$, percentage predicted forced expiratory volume in one second; $FVC_{\%pred}$, percentage predicted forced vital capacity; *t*VLL, total vertical length of the lung; *u*VLL, upper vertical length of the lungs; *l*VLL, lower vertical length of the lung; %LAA, percentage low-attenuation area.

Supplementary Table

Supplementary Table 3. Characteristics of patients with secondary IPs

	Secondary IPs, n=240	IIPs, n=280	Controls, n=280
Age	64 (24-87)	70 (36-92)*	71.0 (24-93)*
Sex, male	113 (47.1)	214 (76.4)*	214 (76.4)*
Body-mass index	22.3 (14.5-32.6)	23.0 (15.6-35.6)**	22.9 (14.7-48.1)**
Spirometry			
%predicted FVC	82.4 (32.6-136.3)	79.3 (28.0-132.8)*	100.0 (79.9-137.1)*
%predicted FEV_1	80.4 (31.2-141.0)	81.4 (34.8-141.1)*	97.1 (77.9-141.4)*
FEV_1/FVC ratio	81.7 (42.1-105.1)	83.2 (47.9-100.0)**	79.0 (70.1-116.3)**

Data presented as median (range) or number (%).
FEV_1, forced expiratory volume in 1 s; FVC, forced vital capacity; IIPs, idiopathic interstitial pneumonias; IPs, interstitial pneumonias.
*$p<0.001$ compared with secondary IPs
**$p<0.050$ compared with secondary IPs

Supplementary Table 4. Comparison of vertical lung length between IIPs and secondary IPs

	All patients		%predicted FVC ≥80%	
	Secondary IPs, n=240	IIPs, n=280	Secondary IPs, n=137	IIPs, n=137
*u*VLL, mm/m	58 (37-80)	59 (43-79)**	59 (40-80)	62 (44-79)**
*l*VLL, mm/m	104 (51-158)	91 (37-130)*	107 (66-141)	98 (59-130)*
*t*VLL, mm/m	163 (100-220)	150 (87-193)*	168 (125-220)	161 (113-193)**
l/*u*VLL ratio	1.79 (0.98-3.64)	1.55 (0.73-2.71)*	1.82 (1.04-2.74)	1.60 (0.95-2.52)*

Data presented as median (range).

IIPs, idiopathic interstitial pneumonias; IPs, interstitial pneumonias; *t*VLL, total vertical length of the lung; *u*VLL, upper vertical length of the lungs; *l*VLL, lower vertical length of the lung.

*$p<0.001$ compared with secondary IPs

**$p<0.050$ compared with secondary IPs
